# Pathogenic Polyglutamine Tracts Are Potent Inducers of Spontaneous Sup35 and Rnq1 Amyloidogenesis

**DOI:** 10.1371/journal.pone.0009642

**Published:** 2010-03-10

**Authors:** Heike Goehler, Anja Dröge, Rudi Lurz, Sigrid Schnoegl, Yury O. Chernoff, Erich E. Wanker

**Affiliations:** 1 Max Delbrueck Center for Molecular Medicine, Berlin, Germany; 2 Max Planck Institute for Molecular Genetics, Berlin, Germany; 3 School of Biology and Institute for Bioengineering and Bioscience, Georgia Institute of Technology, Atlanta, Georgia, United States of America; Brigham and Women's Hospital, Harvard Medical School, United States of America

## Abstract

The glutamine/asparagine (Q/N)-rich yeast prion protein Sup35 has a low intrinsic propensity to spontaneously self-assemble into ordered, β-sheet-rich amyloid fibrils. In yeast cells, *de novo* formation of Sup35 aggregates is greatly facilitated by high protein concentrations and the presence of preformed Q/N-rich protein aggregates that template Sup35 polymerization. Here, we have investigated whether aggregation-promoting polyglutamine (polyQ) tracts can stimulate the *de novo* formation of ordered Sup35 protein aggregates in the absence of Q/N-rich yeast prions. Fusion proteins with polyQ tracts of different lengths were produced and their ability to spontaneously self-assemble into amlyloid structures was analyzed using *in vitro* and *in vivo* model systems. We found that Sup35 fusions with pathogenic (≥54 glutamines), as opposed to non-pathogenic (19 glutamines) polyQ tracts efficiently form seeding-competent protein aggregates. Strikingly, polyQ-mediated *de novo* assembly of Sup35 protein aggregates in yeast cells was independent of pre-existing Q/N-rich protein aggregates. This indicates that increasing the content of aggregation-promoting sequences enhances the tendency of Sup35 to spontaneously self-assemble into insoluble protein aggregates. A similar result was obtained when pathogenic polyQ tracts were linked to the yeast prion protein Rnq1, demonstrating that polyQ sequences are generic inducers of amyloidogenesis. In conclusion, long polyQ sequences are powerful molecular tools that allow the efficient production of seeding-competent amyloid structures.

## Introduction

Amyloids are fibrillar, highly ordered protein aggregates with a typical cross β-sheet structure [1]. They appear in cells as well as in the extracellular space and are often associated with protein misfolding disorders, including neurodegenerative diseases such as Alzheimer's disease, Huntington's disease and prion disease [2], [3]. Functionally, amyloids are involved in numerous physiological processes such as information transfer in neurons, melanosome biogenesis or modulation of translation termination [4]. Although pathogenic and non-pathogenic amyloid assembly are pervasive biological processes, we still lack a comprehensive understanding of the molecular mechanisms that lead to the spontaneous formation of amyloid.

There are striking similarities in the aggregation behaviour of different amyloidogenic peptides and proteins [1]. In the initial phase of amyloidogenesis, aggregation-prone monomers relatively slowly form soluble oligomers [5]. These earliest aggregation species, visible by electron and atomic force microscopy, are small bead-like structures, also described as amorphous protein aggregates [6]. Amyloidogenic oligomers are biochemically and biophysically not very well defined and are currently thought to cause cellular toxicity in numerous amyloid diseases [7]. Over time, they transform into larger aggregate species with a more fibrillar morphology, often termed “protofibrils” [8]. Protofibrils are better defined with regard to size and biological activity and are also highly toxic for mammalian cells [9]. Finally, protofibrils self-assemble efficiently into large mature amyloid fibrils, perhaps through association of monomers or oligomers, which is often accompanied by a structural reorganisation of aggregates [1].

Currently, the molecular mechanisms behind the initiation of amyloid self-assembly are largely unclear. On the one hand, it is thought that oligomer formation is driven by relatively unspecific protein-protein interactions between monomers resulting in undefined, disordered structures [10]. On the other hand, experimental evidence showed the earliest detectable oligomer species to be fairly distinctive structures, suggesting that they are formed by specific protein-protein interactions [11]. In any case, the process is slow and inefficient, with a significant entropic barrier that is mainly controlled by the concentration of monomers [12].

A wide range of factors has been reported to influence spontaneous amyloid assembly [13]. Extrinsic factors are, e.g., the physico-chemical properties of the cellular environment of polypeptide chains (pH, temperature, ionic strength, protein concentration) or molecular chaperones inhibiting or promoting aggregation by directly binding to polypeptide chains [14]. Intrinsic factors are properties of polypeptides, such as charge, hydrophobicity, patterns of polar and non-polar amino acids and the ability to adopt specific secondary structures [15]. In the case of globular proteins, the propensity to spontaneously form amyloid structures is inversely related to the stability of their native states [13]. A large number of proteins that assemble into amyloid, however, are at least partially unfolded under physiological conditions [16].

Experimental evidence has been provided that a high content of the polar amino acids glutamine (Q) and asparagine (N) leads to an increased tendency in proteins to spontaneously form amyloids, implicated in human neurodegeneration and non-Mendelian inheritance of prions in yeast [17]. Several neurodegenerative diseases including Huntington's disease and a variety of spinocerebellar ataxias are caused by a pathogenic expansion of CAG codons in disease genes leading to the production of proteins with elongated polyglutamine (polyQ) tracts. These proteins form inclusion bodies in affected neurons in patient brains that correlate with disease progression and toxicity [2]. Pathogenic polyQ tracts in the Huntington's disease protein, huntingtin, have been shown to stimulate the assembly of amyloid fibrils *in vitro and in vivo* [18], [19], suggesting a function as aggregation-promoting sequences driving amyloidogenesis.

Glutamine/asparagine (Q/N)-rich regions are a characteristic feature of the yeast prion proteins Sup35, Rnq1, Ure2 and Swi1 [17], [20]. In these proteins, the Q/N-rich sequences, as part of prion domains (PrDs), are critical for spontaneous self-assembly of ordered amyloid fibrils and the appearance of defined yeast phenotypes [21]. Previous studies have shown that prions with Q/N-rich regions can facilitate the assembly of polyglutamine aggregates in yeast cells [22]. Glutamine-rich sequences exist in a wide range of proteins across numerous species, suggesting that they have important physiological functions [23]. This is supported by bioinformatic studies, indicating that glutamine-rich domains in proteins are conserved and can undergo positive selection [24]. More specifically, they have been implicated as facilitators of protein complex formation [23], however, their physiological roles are largely unclear.

The structural basis of polyQ-mediated protein aggregation is believed to be the formation of “polar zippers”, in which β-sheets are stabilised by hydrogen bonds between polar amino acids [25]. Once monomers are joined by hydrogen bonds and a significant number of stable protein complexes, so-called seeds, have appeared, amyloid fibrils are formed efficiently by a nucleation-dependent process [26]. Whether polar zipper formation involving Q/N-rich regions in proteins is indeed critical for the initiation of amyloidogenesis, however, is currently unknown. Interestingly, proteins with aggregation-promoting Q/N-rich domains such as the yeast prion protein Sup35 have a relatively low intrinsic propensity to self-assemble [27]. In yeast cells, insoluble Sup35 protein aggregates appear *de novo* at very low frequency. Spontaneous formation of Sup35 aggregates occurs efficiently only at high protein concentrations and is greatly facilitated by the presence of preformed Q/N-rich, seeding-competent nuclei derived from other proteins [28]. This suggests that the aggregation-prone Q/N-rich PrD in the full-length protein is not sufficient to promote spontaneous Sup35 aggregation.

In this study, we have investigated whether polyQ sequences and their ability to form polar zippers can be employed to initiate the amyloidogenesis of soluble Q/N-rich prion proteins such as Sup35. Fusion proteins of Sup35 or its N-terminal PrD with polyQ tracts of different lengths were produced *in vitro* and *in vivo*. Subsequently, the spontaneous formation of stable protein aggregates was analysed using biochemical as well as cell biological methods. Our data indicate that only long, pathogenic polyQ tracts (≥54 glutamines) are potent inducers of Sup35 amyloid polymerisation. PolyQ-stabilised Sup35 amyloids are permanently maintained in yeast cells and form independently of the biological activity of the yeast chaperone Hsp104 or other Q/N-rich yeast prions. Similar results were obtained when polyQ-tagged fusions of the prion protein Rnq1 were systematically analyzed in yeast cells.

## Results

### Pathogenic PolyQ Tracts Stimulate the Self-Assembly of Seeding-Competent PrD Fibrils *In Vitro*

First, we examined whether polyQ tracts of different lengths influence the amyloidogenesis of the Sup35 PrD using an *in vitro* aggregation assay. Formation of SDS-insoluble PrD aggregates was quantified by filtration and immunoblotting [26]. Soluble fusion proteins with an N-terminal maltose binding protein (MBP), a Q/N-rich PrD of Sup35 (1-123 aa) and C-terminal huntingtin-derived polyQ tracts with 19, 55 or 89 glutamines were produced in *E. coli* and purified under native conditions by affinity chromatography. The MBP-tag, which can be removed from the fusion proteins by factor Xa cleavage, allows affinity purification and increases the solubility of the fusions. In contrast, the polyQ sequences at the C-terminus are thought to stimulate amyloidogenesis of the Q/N-rich PrD fragments by polar zipper formation. The PrD was previously shown to slowly form amyloid in cell-free and cell-based assays [29], [30].

**Figure 1:**
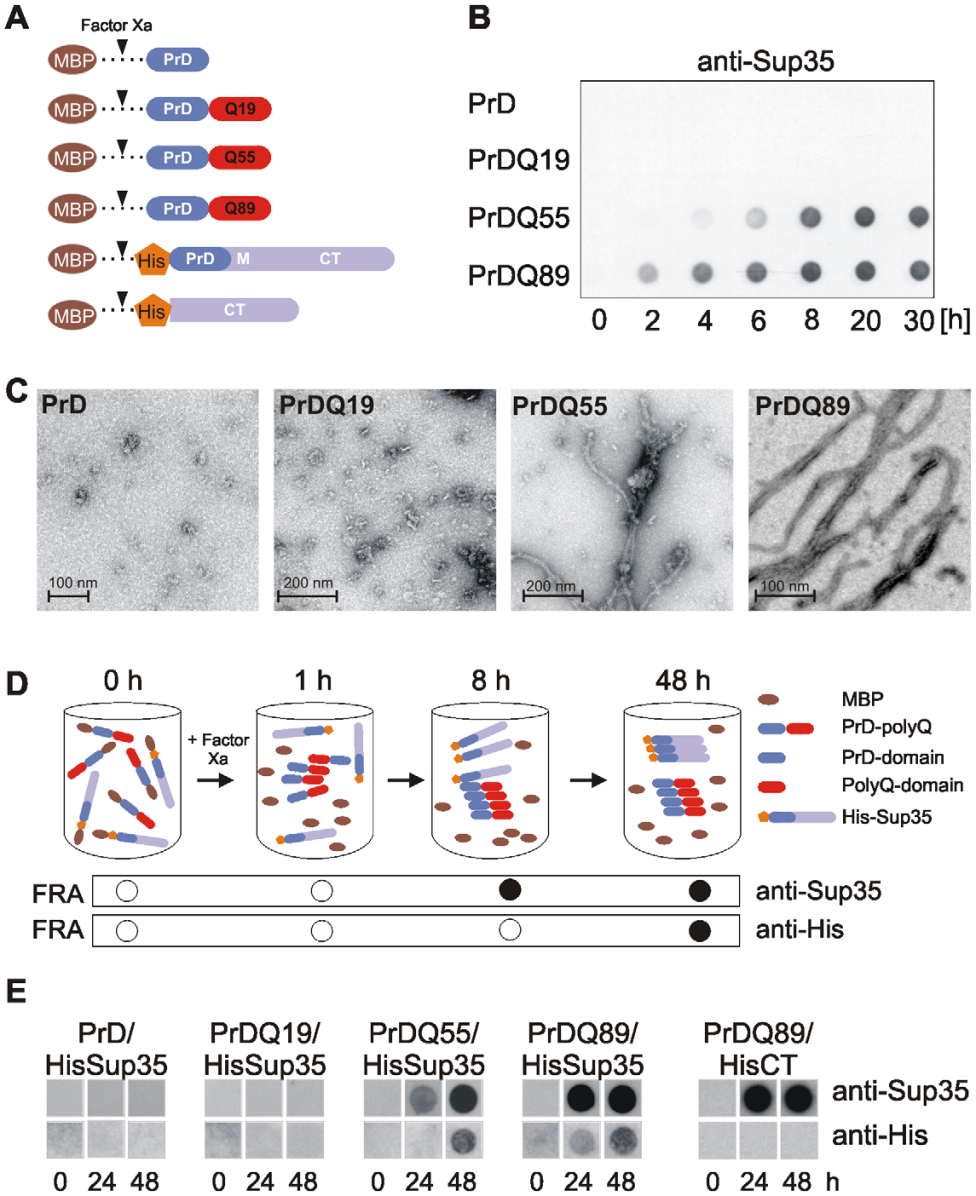
PrD-polyQ fusion proteins form seeding-competent amyloid structures *in vitro*. (A) Schematic representation of MBP fusion proteins with polyQ tracts of different lengths. (B) Time-resolved analysis of polyQ-mediated aggregation of PrD fusions by FRA. SDS-resistant protein aggregates retained on filter membranes were detected using an anti-Sup35 antibody. (C) Electron micrographs of polyQ-containing PrD protein aggregates. MBP fusions were digested with factor Xa without agitation at 37°C for 24h, stained with 1% uranyl acetate and visualised using a Philips CM100 transmission electron microscope. (D) Schematic description of an *in vitro* seeding assay. The formation of seeding-competent polyQ-containing PrD protein aggregates after cleavage of MBP fusion proteins with factor Xa and the subsequent conversion of HisSup35 protein from the soluble to the amyloidogenic state is shown. SDS-stable protein aggregates were monitored by FRA using specific antibodies. (E) Analysis of HisSup35 aggregate formation by FRA. MBP-PrD-polyQ fusion proteins together with MBP-HisSup35 or MBP-HisCT were incubated with factor Xa at 37°C for 24 and 48 h. Aggregates retained on filter membranes were detected using anti-His and anti-Sup35 antibodies.

To initiate spontaneous amyloid assembly, equal amounts of fusion proteins MBP-PrD, -PrDQ19, -PrDQ55 and -PrDQ89 (Fig. 1A) were digested with factor Xa at 37°C and formation of SDS-stable protein aggregates was monitored over time by filter retardation assay (FRA). We observed that the proteins PrDQ55 and PrDQ89 efficiently form SDS-stable protein aggregates after an incubation period of 2–6 h, while the proteins PrD and PrDQ19 under the same conditions do not (Fig. 1B). This indicates that long (≥55 glutamines), but not short polyQ sequences (19 glutamines) are potent inducers of PrD aggregation *in vitro*. The time course analysis also revealed that the PrDQ89 protein aggregates faster than the PrDQ55 protein (Fig. 1B), indicating that initiation of amyloidogenesis is polyQ-length dependent.

To determine whether the SDS-stable polyQ-containing PrD aggregates observed by FRA are fibrillar amyloids, aggregation reactions were analysed by electron microscopy (Fig. 1C). Numerous clusters of large fibrils with a length of 0.2–3 µm and a diameter of about 10 nm were observed with the PrDQ89 or the PrDQ55 fusion proteins, indicating that the long polyQ tracts mediate the self-assembly of typical amyloid structures. In comparison, such structures were not detected with the proteins PrDQ19 and PrD (Fig. 1C). With these proteins, however, smaller, mostly non-fibrillar particles of varying size and shape were frequently observed, indicating that the proteins PrDQ19 and PrD self-assemble into oligomeric structures under these conditions that might be precursors of mature amyloids [31].

To investigate whether the insoluble PrD-polyQ protein aggregates are seeding-competent structures that can stimulate the amyloidogenesis of other soluble Q/N-rich proteins, an *in vitro* seeding assay was established (Fig. 1D). The proteins MBP-PrD, -PrDQ19, -PrDQ55 or -PrDQ89 were incubated together with factor Xa and a soluble MBP-HisSup35 fusion protein; formation of SDS-stable HisSup35 aggregates was monitored after incubation for 24 and 48 h at 37°C by FRA using a monoclonal anti-His antibody. We found that the proteins PrDQ55 and PrDQ89, which efficiently form amyloid structures *in vitro* (Fig. 1B), promote HisSup35 aggregation (Fig. 1E), while the proteins PrD and PrDQ19, which are soluble under these conditions, do not. This indicates that the SDS-stable amyloids formed by PrD-polyQ fusions are seeding-competent structures that can stimulate the polymerisation of soluble Sup35 protein with a homologous Q/N-rich PrD domain.

As previous studies have demonstrated that the PrD in Sup35 is critical for the self-assembly of amyloid fibrils [30], we also examined whether a His-tagged N-terminally truncated Sup35 protein (HisCT, Fig. 1A) can be converted into SDS-stable aggregates. As shown in Fig. 1E, the HisCT fragment (254–685 aa) was not converted from the soluble into the aggregated state by PrDQ89 amyloids, supporting previous studies that the N-terminal Q/N-rich region in Sup35 is critical for aggregation [32], [33].

### Pathogenic PolyQ Tracts Initiate the Self-Assembly of Seeding-Competent PrD and Sup35 Aggregates in the Absence of Q/N-Rich Rnq1 Prions

**Figure 2:**
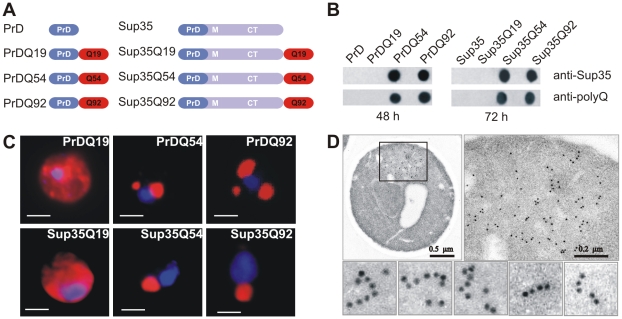
PrD and Sup35 fusion proteins with pathogenic polyQ tracts form amyloid structures *in vivo*. (A) Schematic representation of PrD- and Sup35-polyQ fusion proteins expressed in GT17 yeast cells. (B) Analysis of SDS-stable PrD and Sup35 protein aggregates with polyQ sequences by FRA. Protein aggregates retained on filter membranes were detected using anti-Sup35 and anti-polyQ antisera. (C) Immunofluorescence micrographs of GT17 yeast cells expressing PrD- and Sup35-polyQ fusion proteins. Cells were fixed, permeabilised, and probed with an anti-polyQ antibody and a Cy3-conjugated anti-rabbit IgG. Nuclei were counterstained with Hoechst 33258. Scale bar, 1µm. (D) Electron micrographs of GT17 cells expressing a Sup35Q54 fusion protein. Cells were immunogold labeled using an anti-polyQ antibody. A cytoplasmic inclusion body with aggregated Sup35Q54 protein was detected by immunogold staining. Rows of gold particles indicate the formation of Sup35Q54 fibrils.

To investigate whether polyQ tracts stimulate the self-assembly of Q/N-rich amyloid structures *in vivo*, PrD-polyQ as well as Sup35-polyQ fusion proteins (Fig. 2A) were expressed in the yeast strain GT17 [*psi* ^−^][*pin* ^−^] [34]. In this strain, endogenous Sup35 is soluble and spontaneous aggregation occurs with an extremely low frequency. Previous studies have also demonstrated that the Q/N-rich prion protein Rnq1 is soluble in GT17 cells. In its prion state, Rnq1, however, is aggregated and promotes the polymerization of Sup35 as well as of other polyQ-containing proteins [22], [28]. Thus, our experimental design was chosen to create unfavourable conditions for spontaneous aggregation of Sup35 and PrD fusion proteins, in order to be able to test the effect of polyQ domains on this process.

The formation of SDS-stable PrD-polyQ aggregates in GT17 cells was analysed by FRA after 48 h of growth using anti-polyQ and anti-Sup35 antibodies. Fig. 2B shows that the proteins PrDQ54 and PrDQ92 spontaneously form SDS-stable protein aggregates, while PrD and PrDQ19 do not, although expressed at similar levels (not shown). This supports the *in vitro* results and indicates that preformed Q/N-rich Rnq1 aggregates are not required for *de novo* amyloidogenesis, when PrD fusions with long polyQ tracts are expressed.

Next, we examined full-length Sup35 proteins with polyQ tracts for their ability to self-assemble into insoluble aggregates. The proteins Sup35, Sup35Q19, Sup35Q54 and Sup35Q92 were overproduced in GT17 cells; aggregation was monitored by FRA (Fig. 2A and B). Full-length Sup35 fusions with pathogenic polyQ sequences (Q54 and Q92) formed SDS-stable protein aggregates, whereas no aggregates were observed with the proteins Sup35 and Sup35Q19. While aggregates were detectable with PrDQ54 and PrDQ92 already after 48 h, aggregates of full-length Sup35-polyQ fusions were observed after 72 h, demonstrating that fusion proteins containing shorter Sup35 fragments that include only PrD (aa 1-123) assemble faster than the full-length Sup35 fusions (aa 1-685).

The results obtained by filter assay were confirmed by immunofluorescence microscopy (Fig. 2C). GT17 cells overexpressing PrDQ54 or PrDQ92 contained large perinuclear inclusion bodies (0.5–1 µm) with insoluble protein aggregates. No such structures were observed in yeast cells overproducing PrDQ19 or PrD (not shown). In the vast majority of PrDQ19 expressing cells, fluorescence was diffusely distributed throughout the cytoplasm. Similar results were obtained when yeast cells expressing Sup35-polyQ fusions were analysed (Fig. 2C).

To investigate the morphology of Sup35-polyQ aggregates, experiments with immunogold labelled antibodies were carried out (Fig. 2D). Immunoelectron microscopy of Sup35Q54 expressing cells repeatedly revealed rows of gold particles, suggesting that at least part of the insoluble Sup35-polyQ aggregates have a fibrillar morphology in yeast.

### Aggregates Formed of PrD- and Sup35-PolyQ Fusions Are Seeding-Competent Structures That Stimulate Endogenous Sup35 Amyloidogenesis *In Vivo*

We next investigated whether insoluble PrD- and Sup35-polyQ protein aggregates formed *in vivo* are seeding-competent structures that can convert endogenous Sup35 protein from the soluble to the insoluble state. Previous studies have demonstrated that aggregation of endogenous Sup35 dramatically influences translation fidelity and stimulates read-through of nonsense codons. This can be monitored with a nonsense mutation in the *ADE1* gene (*ade 1-14*) that permits growth of yeast cells on medium lacking adenine (SD-Ade) as a result of Sup35 aggregation [35], [36]. In GT17 cells, endogenous Sup35 protein is soluble; however, it might covert into insoluble aggregates when seeding-competent PrD- or Sup35-polyQ proteins are expressed.

**Figure 3:**
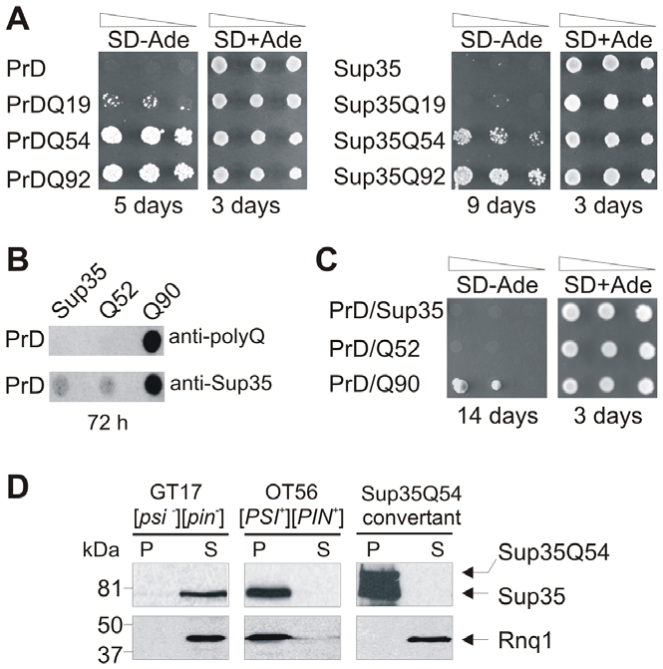
Formation of PrD- and Sup35-polyQ amyloids stimulates endogenous Sup35 aggregation in yeast. (A) Expression of polyQ-containing PrD and Sup35 fusion proteins in [*psi^−^*][*pin^−^*] GT17 yeast cells induces the spontaneous appearance of ADE+ colonies on SD-Ade plates. (B) Analysis of cell lysates of GT17 cells co-expressing the proteins PrD/Sup35, PrD/Q52 or PrD/Q90 by FRA using anti-Sup35 and anti-polyQ antisera. (C) Growth assays of [*psi^−^*][*pin^−^*] GT17 cells co-expressing the proteins PrD/Sup35, PrD/Q52 or PrD/Q90 on SD-Ade plates. (D) Analysis of endogenous Sup35 and Rnq1 protein aggregation in GT17 cells overexpressing Sup35Q54 by centrifugation assays. Proteins were detected by Western blotting using anti-Sup35 and anti-Rnq1 antibodies. P, pellet fraction; S, supernatant fraction.

Serial dilutions of GT17 yeast transformants expressing the different PrD-polyQ fusions were spotted onto SD-Ade plates and formation of ADE+ colonies was examined after an incubation period of 5 days. We found that yeast cells overproducing the proteins PrD or PrDQ19 only very rarely formed ADE+ colonies, while PrDQ54 or PrDQ92 expressing cells formed such colonies with high frequency (Fig. 3A). This indicates that the PrD-polyQ protein aggregates that are observed after 48 h (Fig. 2B) are seeding-competent structures that can promote the aggregation of soluble, endogenous Sup35 protein.

A similar result was obtained when GT17 cells expressing full-length Sup35-polyQ proteins were spotted onto SD-Ade selective plates (Fig. 3A). However, formation of ADE+ colonies was slower with Sup35Q54 or Sup35Q92 than with the PrDQ54 or PrDQ92 fusion proteins. This indicates that the rate of polyQ-mediated aggregation of target proteins correlates with the rate of *de novo* appearance of ADE+ colonies. The faster the PrD-polyQ protein aggregates are formed in GT17 cells, the faster the endogenous Sup35 protein is converted into the amyloidogenic state. The same applies to the full-length Sup35-polyQ fusion proteins. The results obtained with the spotting assay were confirmed in a plating assay that allows a quantification of ADE+ conversion frequencies [37] (Table 1).

**Table 1:** *De novo* appearance of ADE+ colonies.

Plasmid	Protein	Conversion rate	Fold increase
pYex2T	-	1.0×10^−5^	1
pYex2T-PrD	PrD	6.9×10^−5^	7
pYex2T-PrDQ19	PrDQ19	1.6×10^−3^	160
pYex2T-PrDQ54	PrDQ54	3.0×10^−2^	3000
pYex2T-PrDQ92	PrDQ92	5.3×10^−2^	5300
pYex2T-Sup35	Sup35	3.0×10^−5^	3
pYex2T-Sup35Q19	Sup35Q19	3.0×10^−5^	3
pYex2T-Sup35Q54	Sup35Q54	4.6×10^−3^	460
pYex2T-Sup35Q92	Sup35Q92	5.9×10^−3^	590
pYex2T-Q52	Q52	1.0×10^−5^	1
pYex2T-Q83	Q83	1.0×10^−5^	1

The frequency of appearance of ADE+ colonies was determined by plating equal quantities of cells onto SD media with and without adenine. After incubation at 30°C for 10 days conversion rates were determined by counting and averaging the colonies obtained in 3–5 independent experiments.

Experimental evidence has been provided previously that insoluble polyQ aggregates *per se* can promote Sup35 aggregation, when both an N-terminal Sup35 and a polyQ fragment are co-expressed in yeast cells [38]. We therefore also tested whether co-expression of the proteins PrD/Sup35, PrD/Q52 and PrD/Q90 in GT17 cells initiates spontaneous aggregation of endogenous Sup35. In PrD/Q90 expressing cells, we found that SDS-stable Sup35 aggregates are formed (Fig. 3B), confirming previous studies that polyQ aggregates *in trans* can stimulate Sup35 polymerization [38]. In cells expressing the other combinations, no insoluble aggregates were detected. Similar levels of expression of recombinant proteins were detected by SDS-PAGE and immunoblotting (Fig. S1).

The results obtained by FRA were also confirmed when cells were spotted onto SD+Ade and SD-Ade plates (Fig. 3C). However, our studies clearly demonstrate that polyQ sequences *in trans* are less potent initiators of endogenous Sup35 aggregation than polyQ sequences *in cis*. While ADE+ colonies were observed after 5 days on SD-Ade plates with cells expressing PrDQ54 or PrDQ92 fusions (Fig. 3A), they were only observed after 14 days with cells co-expressing the proteins PrD/Q90 (Fig. 3C).

We also examined whether the ADE+ convertants that grow on SD-Ade plates indeed contain aggregated, endogenous Sup35 protein. 10 ADE+ colonies from a Sup35Q54 expressing GT17 strain were randomly picked and analysed for insoluble Sup35 protein using a centrifugation assay [39]. In all cases, endogenous Sup35 protein was detected in the pellet fraction, indicating that formation of polyQ-containing protein aggregates converts endogenous Sup35 protein from the soluble to the aggregated state. A representative centrifugation experiment is shown in Fig. 3D. The randomly chosen ADE+ convertant strain contains aggregated Sup35Q54 fusion protein (∼90 kDa) as well as endogenous Sup35 protein (∼75 kDa). The [*PSI* ^+^][*PIN* ^+^] control strain OT56 also contains aggregated endogenous Sup35 protein. However, the protein was soluble in [*psi* ^−^][*pin* ^−^] GT17 cells. Similar results were obtained when ADE+ convertants expressing the proteins Sup35Q92, PrDQ54 or PrDQ92 were analysed by centrifugation assays (data not shown).

Finally, using centrifugation assays we investigated whether Sup35Q54 overexpression promotes the aggregation of endogenous Rnq1, an aggregation-prone Q/N-rich prion protein [28], [40], which was shown previously to have the potential to stimulate spontaneous Sup35 polymerization. We found that Rnq1 was soluble in Sup35Q54 expressing as well as in GT17 [*psi* ^−^][*pin* ^−^] control cells, while it was insoluble in [*PSI* ^+^][*PIN* ^+^] cells (Fig. 3D). This indicates that production of Sup35-polyQ fusion proteins efficiently stimulates endogenous Sup35 but not Rnq1 aggregation.

### Spontaneous Aggregation of Sup35-PolyQ Proteins Is Not Prevented in ΔHSP104 Cells

Previous studies have demonstrated that self-assembly of seeding-competent Sup35 aggregates *in vitro* critically depends on the activity of the molecular chaperone Hsp104 [35]. Therefore, we investigated whether spontaneous polyQ-mediated Sup35 aggregation is influenced by loss of Hsp104 function.

**Figure 4:**
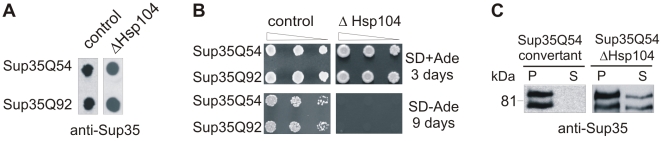
Loss of Hsp104 activity does not prevent the formation of Sup35-polyQ aggregates in yeast. (A) Analysis of Sup35Q54 and Sup35Q92 aggregation in OT78 cells lacking a functional Hsp104 protein (ΔHsp104) by FRA. Protein aggregates retained on filter membranes were detected using an anti-Sup35 antibody. Cell extracts prepared from GT17 cells containing a functional Hsp104 protein (control) were also examined. (B) Growth assays of GT17 and OT78 cells expressing the proteins Sup35Q54 or Sup35Q92 on SD-Ade selective plates. (C) Analysis of Sup35Q54 and endogenous Sup35 aggregation in GT17 and OT78 cells by centrifugation assay. Proteins were detected by Western blotting using an anti-Sup35 antibody. P, pellet fraction; S, supernatant fraction.

Plasmids encoding the proteins Sup35Q54 or Sup35Q92 were transformed into the yeast strain OT78 [*psi* ^−^][*pin* ^−^] lacking the functional Hsp104 protein (ΔHSP104) and formation of SDS-stable, insoluble protein aggregates was analysed by FRA. We observed that the proteins Sup35Q54 or Sup35Q92 readily formed insoluble aggregates in the absence of Hsp104 (Fig. 4A), indicating that loss of Hsp104 activity does not prevent polyQ-mediated Sup35 aggregation *in vivo*.

In a further experiment, OT78 cells expressing the proteins Sup35Q54 or Sup35Q92 were spotted onto SD-Ade selective plates. As shown in Fig. 4B, ADE+ yeast colonies were not observed with the ΔHSP104 OT78 yeast strain, while such colonies were readily detectable with the GT17 strain expressing a functional Hsp104 protein (Fig.3A, data not shown). Thus, loss of Hsp104 function does not prevent Sup35-polyQ amyloidogenesis but seems to prevent endogenous Sup35 aggregation, which is normally induced by Sup35Q54 and Sup35Q92 aggregates in GT17 cells (Fig. 3A).

To investigate this result in more detail, OT78 and GT17 yeast cells overexpressing the fusion protein Sup35Q54 were next analyzed by centrifugation assays. We found that in OT78 cells a small fraction of the proteins Sup35Q54 (∼90 kDa) and endogenous Sup35 (∼75 kDa) is soluble, while both proteins are completely insoluble in GT17 cells (Fig. 4C). This indicates that deletion of the *HSP104* gene increases the solubility of Sup35Q54 and endogenous Sup35 in OT78 cells. We suggest that the appearance of soluble, functional Sup35 protein leads to improved translation termination, which prevents the appearance of ADE+ colonies (Fig. 4B). In strong contrast, in GT17 cells, which exclusively contain Sup35Q54 and Sup35 aggregates, a nonsense suppression phenotype is observed.

### Insoluble Sup35-PolyQ Aggregates Are Required for Initiation of Sup35 Aggregation but Not for Prion Propagation

**Figure 5:**
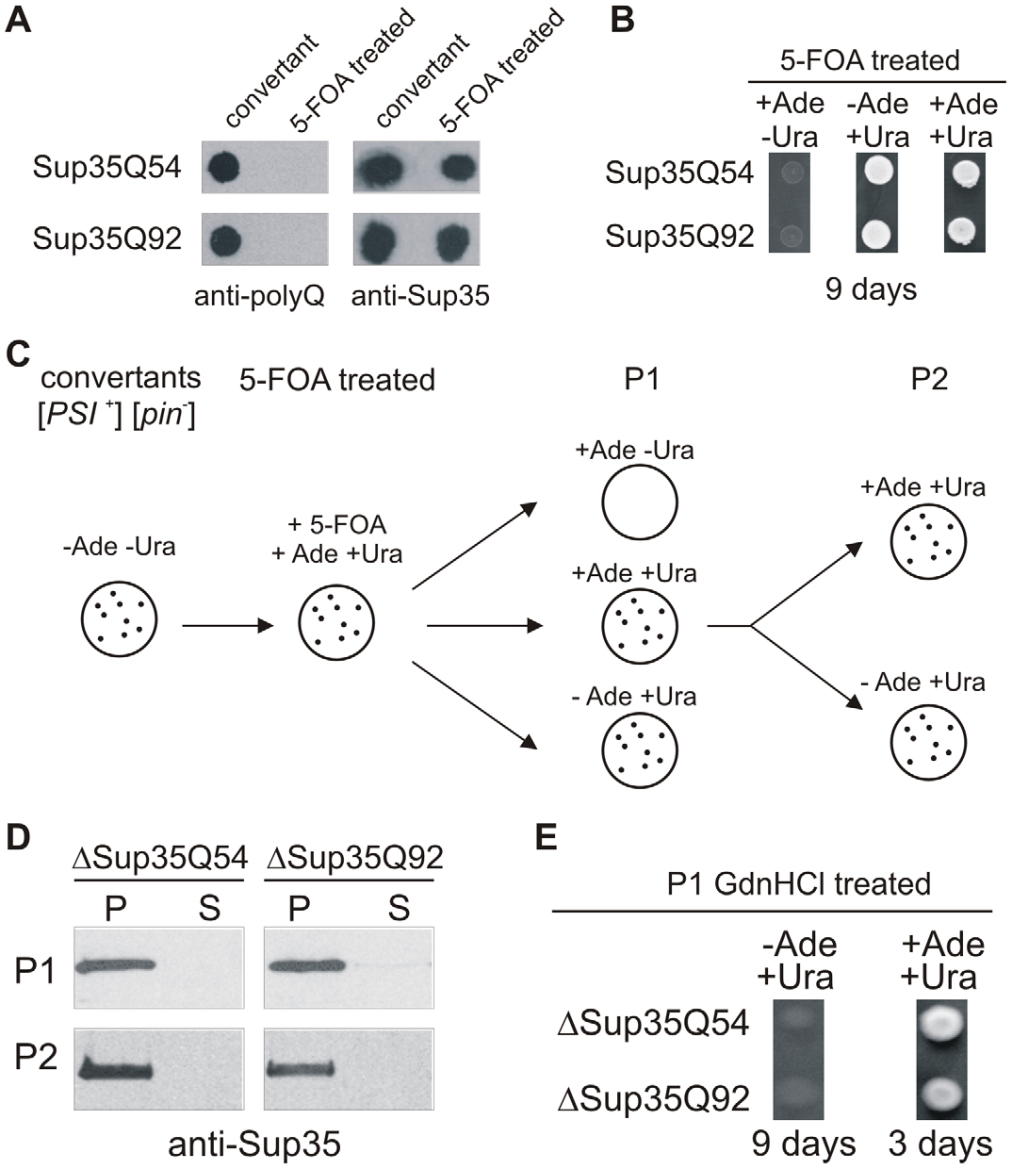
Sup35-polyQ aggregates are not required for propagation of endogenous Sup35 aggregates. (A) Analysis of protein extracts prepared from 5-FOA treated yeast cells by FRA. ADE+ GT17 cells expressing the proteins Sup35Q54 or Sup35Q92 were plated onto 5-FOA medium in order to eliminate *URA3* expression plasmids. SDS-stable aggregates retained on filter membranes were detected using anti-Sup35 and anti-polyQ antibodies. (B) Growth assays of 5-FOA treated GT17 cells on SD-Ade plates. 5-FOA treated yeast cells showed growth on SD-Ade plates, indicating that they contain insoluble, endogenous Sup35 aggregates. (C) Schematic representation of growth assays with 5-FOA treated yeast cells on different selective plates. Yeast cells were grown for many generations on non-selective media in order to investigate the propagation of insoluble Sup35 aggregates. (D) Analysis of Sup35 aggregation in 5-FOA treated yeast cells lacking Sup35-polyQ fusion proteins (ΔSup35Q54 and ΔSup35Q92) by centrifugation assays. P, pellet fraction; S, supernatant fraction. (E) GdnHCl treatment of 5-FOA treated yeast cells (P1) lacking the proteins Sup35Q54 and Sup35Q92. After GdnHCl treatment P1 cells were unable to grow on SD-Ade plates, indicating that they do not contain insoluble Sup35 aggregates.

To investigate whether insoluble Sup35-polyQ aggregates are necessary for propagation of endogenous Sup35 aggregates, we produced yeast cells that do not contain such structures. ADE+ GT17 cells harbouring episomal *URA3* plasmids for the production of Sup35Q54 or Sup35Q92 were plated onto 5-FOA selective medium, and cells lacking the expression plasmids were selected [41]. The resulting yeast cells were then analyzed by FRA whether they contain insoluble Sup35-polyQ or Sup35 aggregates. We observed that the 5-FOA selected yeast cells contain SDS-stable Sup35 protein aggregates, while Sup35Q54 or Sup35Q92 aggregates were not detected (Fig. 5A). Spotting of 5-FOA treated yeast cells onto SD-Ade and SD+Ade plates confirmed these results (Fig. 5B). Yeast cells lacking Sup35-polyQ aggregates formed ADE+ colonies on SD-Ade plates with similar frequency than yeast strains expressing the proteins Sup35Q54 or Sup35Q92 (Fig. 5B, data not shown). Thus, the formation of Sup35Q54 or Sup35Q92 aggregates is critical for the initiation of endogenous Sup35 aggregation (Fig. 3A) but not for propagation of insoluble Sup35 aggregates.

To study whether the insoluble Sup35 aggregates in 5-FOA treated cells are efficiently passed on from mother to daughter cells, we determined the frequency of formation of ADE+ colonies on SD-Ade and SD+Ade plates after growing cells for many generations (passage 1 and 2) on non-selective plates (Fig. 5C). We observed that passage 1 and 2 cells formed ADE+ colonies with the same frequency (∼5×10^−2^), indicating that the insoluble Sup35 aggregates are stably propagated and efficiently passed on from mother to daughter cells. These results were also confirmed by centrifugation assay (Fig. 5D).

Finally, we investigated whether 5-FOA treated cells lacking polyQ-containing Sup35 aggregates (P1 cells) can be cured of endogenous Sup35 aggregates by guanidine hydrochloride (GdnHCl) treatment [42]. We found that P1 cells after GdnHCl treatment were unable to grow on SD-Ade plates, while untreated cells efficiently formed ADE+ colonies under such conditions (Fig. 5E, data not shown). Thus, our data strongly indicate that polyQ-mediated Sup35 aggregation induces [*PSI* ^+^] yeast prions, which can be cured by GdnHCl treatement.

### Pathogenic PolyQ Tracts Stimulate the Self-Assembly of Insoluble, Seeding-Competent Rnq1 Protein Aggregates

**Figure 6:**
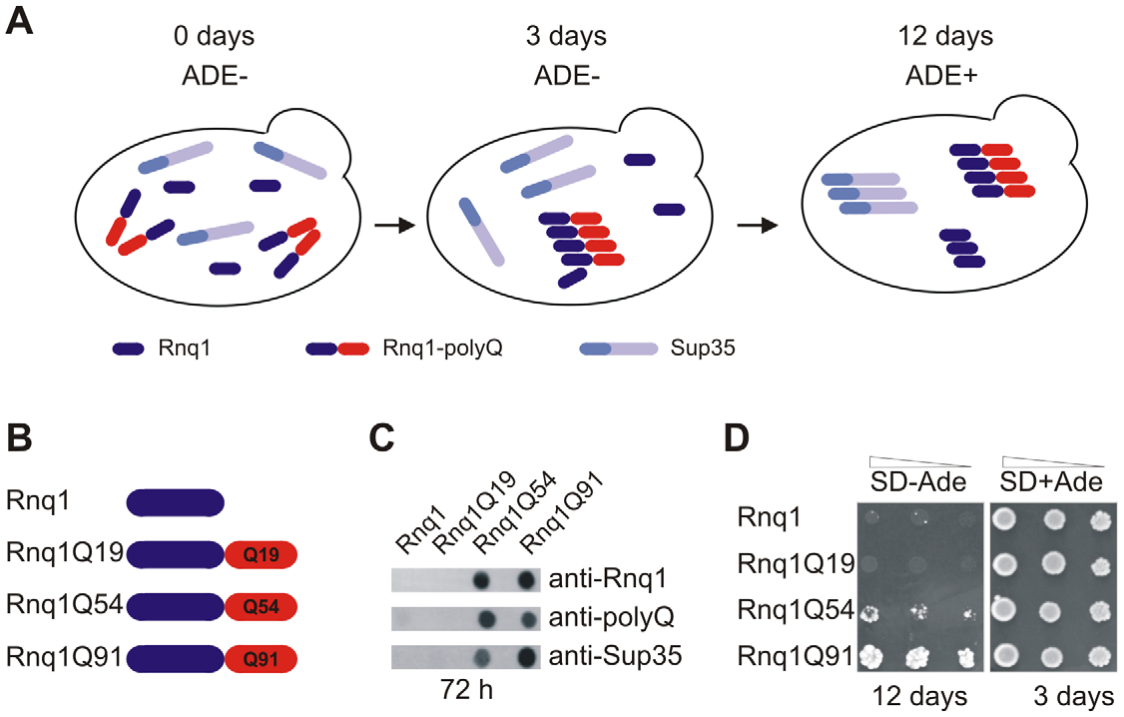
Aggregates of Rnq1-polyQ fusion proteins stimulate the polymerization of endogenous Sup35 protein. (A) Cartoon illustrating that formation of seeding-competent Rnq1-polyQ aggregates, which promote polymerization of endogenous Rnq1 and Sup35. Spontaneous assembly of Sup35 protein aggregates is monitored by growth assays on SD-Ade plates. (B) Schematic representation of Rnq1-polyQ fusion proteins. (C) Analysis of cell extracts prepared from yeast strains expressing polyQ-containing Rnq1 proteins by FRA. (D) Growth assays on SD-Ade selective plates. GT17 [pYex2T-Sup35] yeast cells overexpressing the fusion proteins Rnq1Q54 or Rnq1Q91 form ADE+ colonies on SD-Ade plates.

To test whether polyQ sequences can be utilised more generally to stimulate spontaneous amyloidogenesis of Q/N-rich proteins, we fused such sequences of different lengths to the C-terminus of the yeast protein Rnq1 [28], [40]. Previous studies have demonstrated that insoluble Rnq1 aggregates stimulate Sup35 amyloidogenesis in yeast when both proteins are overproduced [28], [34]. Thus, polyQ-mediated formation of Rnq1 aggregates in Sup35 overexpressing GT17 cells should stimulate Sup35 amyloidogenesis and induce the *de novo* appearance of ADE+ convertants (Fig. 6A).

To test this hypothesis, expression plasmids encoding the fusion proteins Rnq1Q19, Rnq1Q54 and Rnq1Q91 were constructed and transformed into Sup35 overexpressing GT17 [pYex2T-Sup35] cells (Fig. 6B). Formation of SDS-resistant protein aggregates was analysed by FRA (Fig. 6C). We observed that the proteins Rnq1Q54 and Rnq1Q91 form SDS-stable aggregates in GT17 [pYex2T-Sup35] cells, while no aggregates were detected with the proteins Rnq1 and Rnq1Q19. This supports the results obtained with the PrD- and Sup35-polyQ fusion proteins and substantiates the hypothesis that aggregtion-promoting polyQ tracts (≥54 glutamines) induce the *de novo* aggregation of Q/N-rich proteins. Strikingly, the filter assays revealed that cell extracts with insoluble Rnq1Q54 or Rnq1Q91 aggregates also contained SDS-stable Sup35 aggregates, indicating that formation of polyQ-tagged Rnq1 aggregates indeed stimulates the aggregation of Sup35, which contains a heterologous Q/N-rich prion domain. Thus, polyQ-mediated aggregation of one Q/N-rich protein can convert another Q/N-rich protein from the soluble to the amyloidogenic state.

To confirm these results, growth assays were performed and serial dilutions of yeast transformants were spotted onto SD-Ade plates. As shown in Fig. 6D, cells overexpressing the proteins Rnq1Q54 and Rnq1Q91 formed ADE+ colonies on SD-Ade plates, while such colonies were only very rarely detected with yeast cells overproducing the proteins Rnq1 or Rnq1Q19. These data indicate that formation of polyQ-tagged Rnq1 protein aggregates can stimulates Sup35 aggregation in yeast.

## Discussion

In this study, we have systematically analysed the spontaneous aggregation of hybrid fusion proteins with Q/N-rich PrDs and polyQ sequences of different lengths using cell-free and cell-based assays. We found that PrD and Sup35 fusions with long, pathogenic polyQ tracts (≥54 glutamines) efficiently self-assemble into seeding-competent, SDS-stable protein aggregates, while fusions with short, non-pathogenic polyQ tracts (19 glutamines) or proteins without a polyQ tag do not. Moreover, we observed that *de novo* amyloidogenesis of PrD- or Sup35-polyQ fusions is largely independent of extrinsic factors such as other Q/N-rich yeast prions, indicating that increasing the content of aggregation-promoting sequences in proteins enhances their tendency to spontaneously self-assemble into insoluble protein aggregates.

Why do long but not short polyQ tracts stimulate spontaneous amyloidogenesis? Recent NMR studies revealed that long polyQ tracts are structurally very similar to short polyQ tracts [43]. This implies that both species should have similar biochemical and biophysical properties and both should be able to stimulate spontaneous amyloid assembly. We suggest that long polyQ sequences are more potent initiators of *de novo* aggregation because they can form hyperstable β-sheets in soluble proteins independently of their folding state. These β-sheets, stabilised by hydrogen bonds, are exposed on the protein surface. In this way, they might be able to associate with β-sheets of long polyQ tracts in other proteins and thereby could form stable aggregation-competent protein complexes. In strong contrast, short polyQ sequences do not form stable β-sheets in soluble proteins. Therefore, the spontaneous assembly of amyloidogenic protein aggregates is unlikely, and extrinsic factors such as molecular chaperones or preformed β-sheet-rich aggregates are required to mediate amyloid formation. Recent structural studies support the hypothesis that short polyQ sequences in huntingtin do not form stable β-sheets [44]. Instead, they are highly flexible and can adopt multiple conformations, including β-helical, random coil and extended loop structures.

Structural studies of polyQ-containing fibrillar aggregates suggest that they consist of cylindrical β-sheets held together by hydrogen bonds between the main chain and the side chain amides [25]. In this model, one turn of β-sheets consists of 20 glutamine residues. It is most likely unstable because it cannot be held together by hydrogen bonds. In strong contrast, a β-sheet structure of two helical turns (∼40 glutamines) is much more stable because it is joined together by hydrogen bonds between amides of successive turns. This is coherent with our results that proteins with long polyQ tracts (≥54 glutamines) form stable amyloid structures much faster than proteins with short a polyQ sequence (19 glutamines). At the same time, a pathogenic threshold of ∼40 glutamines was observed in a large number of polyQ diseases like Huntington's disease or spinocerebellar ataxias [45].

There have been several studies on long polyQ tracts and their ability to form stable β-sheets in soluble protein molecules. Using CD spectroscopy, Nagai et al. (2007) [46] e.g. have demonstrated that proteins with polyQ tracts of 62 glutamines efficiently adopt a β-sheet conformation, while such a structure was not observed with polyQ tracts of 19 glutamines. Interestingly, these authors also showed that β-sheet formation precedes spontaneous aggregation, indicating that it is critical for initiation of protein polymerization. Structural changes in proteins with long polyQ tracts that mediate aggregation were also observed using fluorescence resonance energy transfer (FRET) and conformation specific anti-polyQ antibodies [47], [48], supporting the results obtained with CD spectroscopy.

**Figure 7:**
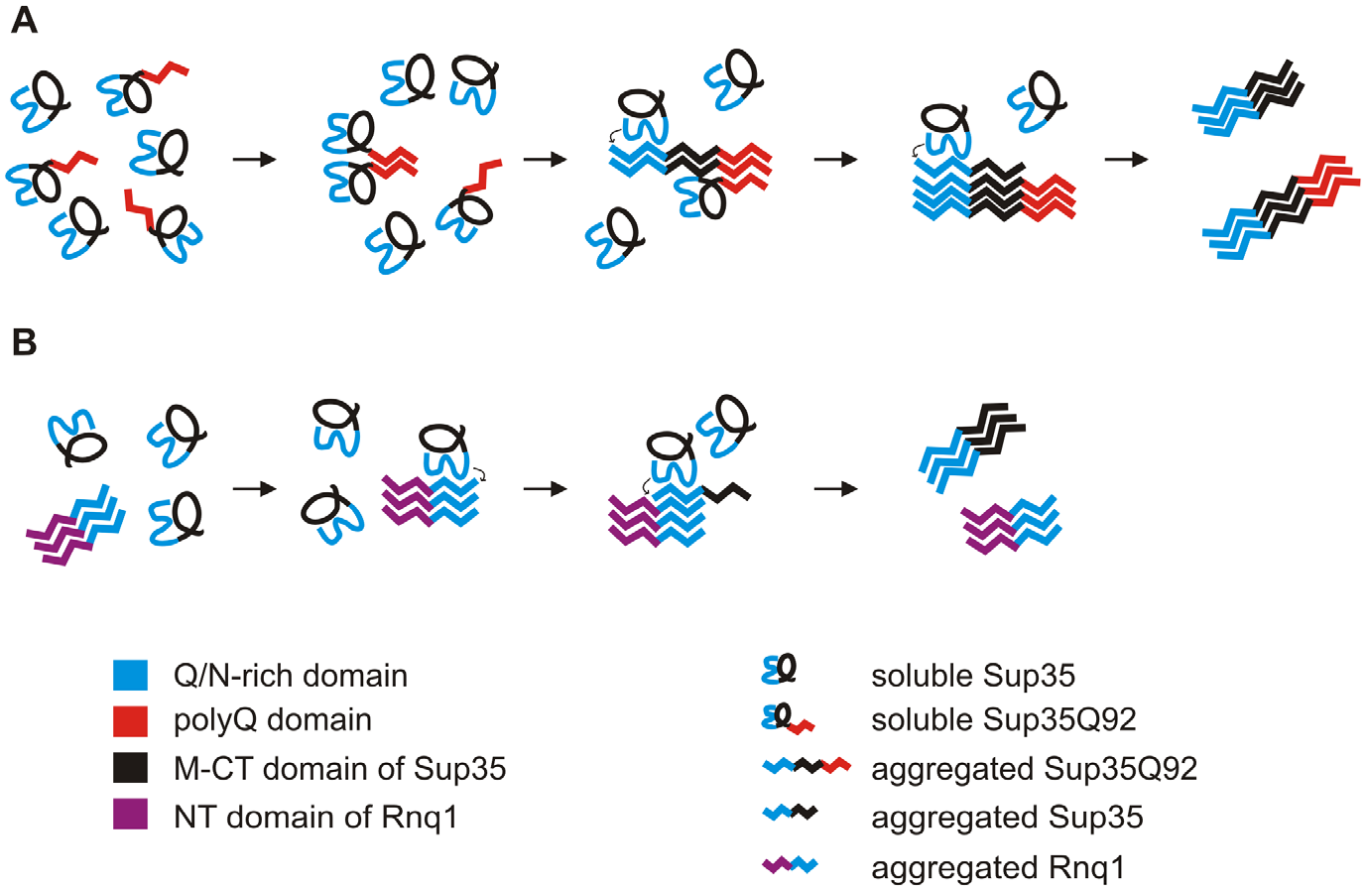
A model for polyQ- and template-mediated Sup35 aggregation in yeast. (A) PolyQ-mediated Sup35 aggregation converts endogenous Sup35 from the soluble into the insoluble state. Soluble Sup35Q92 monomers are joined together by polyQ-mediated polar zipper formation leading to the assembly of insoluble β-sheet-rich protein aggregates. These structures then function as templates for the polymerization of endogenous Sup35 molecules. Sup35Q92-mediated Sup35 aggregation is triggered by the Q/N-rich PrDs present in both proteins. Sup35 aggregation is accompanied by conformational changes in the Q/N-rich PrD leading to the formation of aggregation-prone β-sheet-rich structures. (B) Template-mediated Sup35 aggregation. Pre-existing β-sheet-rich Rnq1 aggregates with an accessible Q/N-rich PrD stimulate the aggregation of soluble Sup35 molecules, which contain a related Q/N-rich PrD. Aggregation is accompanied by conformational changes in the Sup35 PrD leading to β-sheet-rich structures.

Previous studies have demonstrated that the N-terminal Q/N-rich PrD drives spontaneous Sup35 aggregation [49]. This process, however, is slow and relatively inefficient, because the Q/N-rich sequence in Sup35 is not easily accessible and needs to be unmasked to allow β-sheet formation [50]. Extrinsic factors such as preformed Q/N-rich protein aggregates with a high β-sheet content are required to stimulate conformational changes in soluble Sup35, leading to spontaneous polymerization of protein molecules [31]. Our results indicate that C-terminal aggregation-prone polyQ tracts with pathogenic lengths reduce the requirement for extrinsic stimulators of Sup35 aggregation. Long polyQ tracts most likely form stable β-sheets in proteins and thereby facilitate the association of soluble protein molecules by polar zippers formation [25]. A model for spontaneous polyQ-mediated Sup35 aggregation in comparison to template-mediated Sup35 polymerization is shown in Fig. 7.

Biochemical and cell biological studies have demonstrated that insoluble Q/N-rich Rnq1 as well as polyQ aggregates are able to convert Sup35 from the soluble into the insoluble state, indicating that template-mediated heterologous seeding is critical for spontaneous Sup35 aggregation [28], [34], [38]. In our studies, we could successfully confirm these results. However, we also observed that long polyQ tracts fused to the C-terminus are more potent initiators of Sup35 polymerization than performed heterogenous protein aggregates. This indicates that amyloid templates with Q/N-rich domains in trans promote polymerization of soluble Sup35 molecules less efficiently than aggregation-prone polyQ sequences in cis.

We assume that polyQ-mediated polar zipper formation in Sup35 fusion proteins leads to enhanced misfolding, protein aggregation and an improved exposure of normally masked Q/N-rich domains. These domains then can associate with homologous domains in soluble Sup35 molecules, leading to conformational changes and spontaneous self-assembly of Sup35 into insoluble aggregates (Fig. 7A).

Our data indicate that polar amino acids of a certain length (≥54 glutamines) are necessary for promoting Sup35 aggregation. However, previous studies have demonstrated that short hydrophobic polypeptide sequences fused to the C-terminus are also able to induce Sup35 polymerization [28], [34]. Thus, besides long polyQ tracts, hydrophobic protein domains also have potential aggregation-promoting functions. However, more detailed studies are necessary to address the mechanism of aggregation initiation of non-polar amino acid sequences.

Our data indicate that the activity of the molecular chaperone Hsp104 is not required for aggregation of Sup35 fusion proteins with pathogenic polyQ tracts. SDS-stable Sup35Q54 aggregates e.g., were readily observed in [*pin* ^−^][*psi* ^−^] cells in the absence of Hsp104 (Fig. 4A), suggesting that endogenous Sup35 should also be efficiently converted from the soluble into the aggregated state. However, growth assays revealed that ΔHSP104 cells expressing Sup35Q54 do not form ADE+ colonies on SD-Ade plates, indicating that endogenous Sup35 remains soluble in these cells. Analysis of cell extracts with centrifugation assays confirmed that in cells lacking Hsp104 activity a small fraction of both Sup35Q54 and endogenous Sup35 remains soluble, indicating that Hsp104 function is critical for efficient aggregation of both proteins. These data support previous studies demonstrating that Hsp104 catalyzes the formation of new Sup35 polymers *in vivo* [51]. Thus, it seems reasonable to speculate that Hsp104 maintains Sup35 aggregates in [*PSI* ^+^] cells not only by severing existing aggregates but also by promoting the formation of new seeding-competent insoluble structures [35], [52], [53].

Recent studies have demonstrated that the Q/N-rich sequence in the PrD domain of Sup35 can be replaced by polyQ sequences without impairing the ability of the protein to form aggregates [49], [54]. However, the polyQ-Sup35 hybrid proteins lacking the Q/N-rich domain were not able to stimulate aggregation of endogenous Sup35. This demonstrates that polyQ tracts do not fully functionally replace Q/N-rich domains in yeast prions. In our studies polyQ tracts were not utilized to substitute the aggregation-promoting function of PrDs. Rather, we intended to examine the spontaneous aggregation of artificial Sup35 fusion proteins containing two potential aggregation-promoting domains such as a Q/N-rich PrD and a polyQ tract.

A large number of neurodegenerative disorders including Huntington's disease are caused by the expansion of polyQ tracts that drive aggregation of the affected proteins. Our data demonstrate that pathogenic polyQ tracts are potent initiators of amyloidogenesis, dramatically accelerating spontaneous protein self-assembly. Pathogenic polyQ sequences are able to escape the cellular control mechanisms and to act as seeding templates for homologous as well as heterologous proteins. This means that the disease proteins in polyQ disorders might also convert unrelated proteins into amyloid structures. Therapeutic strategies for polyQ diseases therefore need to focus on small molecules that target pathogenic but not non-pathogenic polyQ sequences. Moreover, they should not influence the normal function of glutamine-rich proteins, which are commonly found in mammalian cells. Our polyQ-mediated aggregation system in yeast could be very useful for the identification of modulators that specifically influence the self-assembly of disease-associated polyQ tracts and trigger neurodegeneration in various diseases.

## Materials and Methods

### Yeast Strains

GT17 (*MAT* **a** *ade1-14 his3-Δ200 leu2-3,112 trp-289 ura3-52* [*psi* ^−^][*pin* ^−^]), OT56 (*MAT* **a** *ade1-14 his3-Δ200 leu2-3,112 trp-289 ura3-52* [*PSI* ^+^][*PIN* ^+^]) and OT78 (*MAT* **a** *ade1-14 his3-Δ200 leu2-3,112 trp-289 ura3-52 hsp104Δ::LEU2* [*psi* ^−^][*pin* ^−^]) are derivatives of the yeast strain 74D-694 [33], [34], [35].

### Plasmids

For protein expression in *Saccharomyces cerevisiae*, the plasmids pYex2T [55], pYex2T/L and pGADcup1 were used. pYex2T is a pYEXbx-based 2µ *URA3 leu2-d* vector (Clontech) containing a copper inducible promoter. pYex2T/L was constructed from pYex2T by deleting the 1.2 kb *BstE*II-*Kpn*I fragment carrying the *leu2-d* sequence. To subclone the promoter and the terminator of pYex2T into pGAD426 (Clontech), a PCR fragment flanked by *SphI* recognition sites was amplified using the primer pair P01/P02 (Table S1). Expression vector pGADcup1 was generated by insertion of this fragment into the *SphI* site of pGAD426. To generate the expression constructs pYex2T-PrD and pYex2T-Sup35, the sequences encoding the PrD domain and full-length Sup35 were PCR amplified using the primer pairs P03/P04 and P03/P05 which carry *Xho*I und *Not*I recognition sites required for cloning of the PCR products in pYex2T. Plasmids pYex2T-PrDQ19, pYex2T-PrDQ54, pYex2T-PrDQ92, pYex2T-Sup35Q19, pYex2T-Sup35Q54 and pYex2T-Sup35Q92 were constructed by introducing htt exon 1 PCR fragments (P06/P07) obtained from pCAG20, pCAG51 and pCAG122 [18] into the plasmids pYex2T-PrD and pYex2T-Sup35 via *NotI* and *EcoI* restriction sites. In addition, these PCR fragments were inserted into the *NotI* and *EcoI* restriction sites of pYex2T to obtain pYex2T-Q19, pYex2T-Q54, and pYex2T-Q92, respectively. To generate pGADcup1-PrD, a PrD fragment flanked by *BamH*I and *EcoR*I restriction sites was isolated from pYex2T-PrD and subcloned into pGADcup1. The plasmid pMAL-c2X (Biolabs) was used for the expression of maltose-binding protein (MBP) target protein fusions with a factor Xa cleavage site. To generate pMAL-c2x-PrD and pMal-c2x-HisSup35, PCR fragments were amplified from pYex2T-Sup35 using the primers P08/P09 or P10/11 and inserted into the *EcoR*I and *BamH*I sites of pMAL-c2X. Using the primer pair P12/13 PCR fragments were amplified from pYex2T-Q19, pYex2T-Q54, and pYex2T-Q92, respectively, and inserted into the *Ps*tI and *Hind*III recognition sites of pMAL-c2x (Biolabs) to obtain the three expression plasmids pMAL-Q19, pMAL-Q55 and pMAL-Q89. pMAL-c2X-HisCT was constructed by inserting a DNA fragment encoding a C-terminal Sup35 (254-685 aa, HisCT) protein into the *Sal*I/*Not*I site of pYex2T. The HisCT fragment was generated by PCR amplification using pYex2T-Sup35 as template and primers P14/P05. To generate constructs for the recombinant expression of PrDQ19, PrDQ55 and PrDQ89 in *E.coli*, DNA fragments were amplified by PCR from the corresponding pYex2T plasmids using the primers P08/P15, and inserted into the *EcoR*I site of pMAL-c2X. The Rnq1 encoding reading frame was PCR amplified from *S. cerevisiae* genomic DNA using the primers P16/P17 and cloned into the *Sal*I/*Not*I sites of pYex2T/L. To construct expression plasmids encoding the Rnq-polyQ fusion proteins, we first inserted htt exon 1 PCR fragments with 19, 54 and 91 CAG repeats into the *Not*I and *EcoR*I sites of pYex2T/L, thereby creating plasmid intermediates. Subsequently, a Rnq1 encoding PCR product amplified from pYex2T/L-Rnq1 with the primers P16/P18 was inserted into the *Sal*I/*Not*I sites of the intermediate plasmids resulting in the plasmids pYex2T/L-Rnq1Q19, pYex2T/L-Rnq1Q54 and pYex2T/L-Rnq1Q91. The sequences of all primers are available online in Table S1.

### Antibodies

The polyclonal antibodies anti-polyQ (anti-CAG53b), anti-Sup35 and anti-Rnq1 have been described elsewhere [18], [37], [40]; the anti-His antibody was purchased from Qiagen.

### Protein Expression and Purification

MBP-PrD, MBP-PrDQ19, MBP-PrDQ54, MBP-PrDQ89, MBP-HisSup35, and MBP-HisCT were expressed in the *E. coli* strain BL21-CodonPlus-RP (Stratagene). The cells were grown at 37°C to an OD_600_ of 0.5 and then induced with 0.3 mM IPTG for 5 h at 30°C. Liquid cultures were harvested by centrifugation at 4,000 g for 20 min. Cell pellets were washed once with PBS and stored at −80°C. To purify the fusion proteins, frozen pellets were thawed on ice, resuspended in 40 ml MBP-buffer [20 mM Tris-HCl pH 7.4, 200 mM NaCl, 5 mM MgCl_2_, EDTA-free protease inhibitor (Roche)] containing 0.5 mg/ml lysozyme and incubated on ice for 30 min. Cell lysis was performed by sonication with a Branson B-450 sonifier (1 min, 100 W), and insoluble components were removed by centrifugation (10,000 g, 30 min 4°C). Supernatants were transferred to 50 ml tubes and incubated with pre-washed amylose beads (BioLabs) for 2 h at 4°C with rotation. Proteins bound to the affinity matrix were packed to empty 10 ml columns (MoBiTec) and washed three times with MBP-buffer. 1.5 ml fractions were eluted with MBP-buffer containing 10 mM maltose and the resulting protein solutions were dialysed against cleavage-buffer (20 mM Tris-HCl pH 8.0, 100 mM NaCl, 8.7% glycerol). Aggregated material was removed by ultracentrifugation (100,000 g, 20 min, 4°C) and aliquots of the clarified protein solutions were frozen in liquid nitrogen and stored at −80°C. Protein concentrations were determined using a Bio-Rad protein assay (Biorad), purity of the samples was monitored by SDS-PAGE and Coomassie staining.

### *In Vitro* Filter Retardation Assay

For *in vitro* aggregation assays, purified fusion proteins were subjected to ultracentrifugation (100,000 g, 20 min) to remove aggregated material. Fusion proteins were diluted to a concentration of 200 ng/µl in digestion buffer [20 mM Tris-HCl, pH 8.0, 100 mM NaCl, 2 mM CaCl_2_] and treated with 0.9 µg of factor Xa in a reaction volume of 90 µl. Samples were incubated at 37°C without agitation. For time course experiments, samples were collected after 0, 2, 4, 6, 8, 20 and 30 h, frozen in liquid nitrogen and stored at −80°C. For further analysis, samples were denatured for 5 min at 95°C in the presence of 2% SDS and 50 mM DTT. Subsequently, 20 µl aliquots were analysed by filter retardation assay (FRA) as described [56]. Aggregated material on filters was detected using anti-polyQ and anti-Sup35 specific antisera. For seeding experiments, 100 ng/µl MBP-HisSup35 or 100 ng/µl MBP-HisSupCT were mixed with 1.4 µg factor Xa and 200 ng/µl of MBP-PrD, MBP-PrDQ19, MBP-PrDQ55 and MBP-PrDQ89, respectively, in a final volume of 90 µl. Samples were incubated for 0, 24, and 48 h before they were heat denaturated and subjected to FRA as described above.

### Culture Conditions

Unless noted otherwise, yeast strains were grown in complete glucose medium (YPD) at 30°C. Selective media lacking adenine and containing glucose as carbon source were used for selection of ADE+ colonies. To remove *URA3* plasmids from yeast cells, SD medium supplemented with uracil and 500 mg/l 5-fluoroorotic acid (5-FOA) was used [41]. For GdnHCl treatment, yeast colonies were passaged three times on YPD plates containing 5 mM GdnHCl, transferred to YPD and then to SD-Ade medium to assay the *ade1-14* nonsense suppressor phenotype [57], [58]. Transformants were grown on synthetic glucose media (SD) selective for plasmid maintenance (e.g. SD-Ura).

### Growth Assays

Single yeast colonies were inoculated in selective SD medium and incubated over night at 30°C. The OD_600_ of cultures was determined and used to adjust the cell densities to a uniform concentration of 5×10^7^ cells/ml. To perform qualitative growth assays, three dilutions (1∶10, 1∶100 and 1∶1000) of yeast cultures were prepared and spotted onto SD media supplemented with or without adenine using a stamp. Subsequently cells were incubated at 30°C for three to twelve days. To perform quantitative growth assays, equal cell numbers were plated onto appropriate SD media (SD-Ade and SD+Ade) and incubated at 30°C for 10 days. The number of colonies was counted and the conversion frequencies was determined by dividing the number of yeast colonies appearing on SD-Ade plates (ADE+phenotype) by the number of yeast colonies appearing on SD+Ade plates.

### *In Vivo* Filter Retardation Assay

Yeast cells overexpressing polyQ-containing Sup35 proteins were grown for 2–3 days in the appropriate selective media. For each assay, ∼5×10^8^ cells were harvested and washed with PBS. Pellets were suspended in 300 µl of denaturation solution containing 2% SDS and 50 mM DTT. After addition of 300 µl glass beads, cell lysis was accomplished by three cycles of freeze-thaw, each followed by 1 min of vigorous vortexing. Cell debris was removed by centrifugation at 3,000 g and supernatants were incubated with 100 U/ml Benzonase (Merck) on ice for 30 min. Subsequently, samples were boiled for 5 min and analysed by FRA using anti-polyQ, anti-Sup35 and anti-Rnq1 antisera.

### Centrifugation Assays

High speed centrifugations were performed as described before [39], with minor modifications. To prepare protein extracts, 50 ml cultures of OT56, GT17 and GT17 cells expressing Sup35Q54 were grown in appropriate selective media for 24 h at 30°C. In each case, 2.5×10^8^ cells were harvested and pellets were washed once in PBS, frozen in liquid nitrogen and subsequently thawed at 50°C. Cells were resuspended in two volumes of UC buffer [25 mM Tris-HCl pH 7.5, 50 mM KCl, 10 mM MgCl_2_, 5% Glycerol, 1% Tween 20, 1 mM PMSF, EDTA-free protease inhibitors (Roche)] and after addition of one volume of glass beads disrupted by vortexing for 3 min at room temperature. Debris was removed by centrifugation (1,000 g, 3 min). 100 µl of the supernatants were subjected to ultra centrifugation (100,000 g, 30 min, 4°C) to precipitate aggregates. The pellets were resuspended in 100 µl of UC buffer and 25 µg total protein was analysed by SDS-PAGE and Western blotting using an anti-Sup35 antiserum.

### Electron Microscopy

To study the formation of fibrillar structures *in vitro*, purified MBP-fusion proteins were subjected to ultracentrifugation (100,000 g, 20 min, 4°C) in order to remove preformed aggregated material. To initiate aggregation, 200 ng/µl MBP-fusion protein was digested with 0.9 µg of factor Xa (Biolabs) in a total volume of 90 µl digest-buffer [20 mM Tris-HCl, pH 8.0, 100 mM NaCl, 2 mM CaCl_2_] at 37°C for 24 h without agitation. For electron microscopy, samples were adsorbed to carbon-coated copper and negatively stained with 1% uranyl acetate. Preparations were viewed using a Philips CM100 transmission electron microscope. To visualise Sup35-polyQ aggregates in yeast cells, a single yeast colony was inoculated in selective SD medium and grown for three days at 30°C. The culture was diluted with YPD medium to an OD_600_ of 0.3 and then incubated for 3 h at 30°C. The culture was supplemented with 0.25% glutaraldehyde and 2% formaldehyde, post-fixed with 1% sodium periodate and embedded in LR White resin. 60 nm sections were immunolabeled with a 1∶400 dilution of anti-htt antibody, followed by a 1∶100 dilution of goat anti-rabbit IgG cross-linked to 10 nm gold particles (British BioCell). Preparations were viewed using a Philips CM100 transmission electron microscope.

### Immunofluorescence Microscopy

A single yeast colony was inoculated in selective SD medium and grown for three days at 30°C. The culture was diluted with YPD medium to an OD_600_ of 0.3 and then incubated for 3 h at 30°C. Cells were washed once with PBS, resuspended in PBS, fixed with formaldehyde at a final concentration of 4% and incubated for 1 h at room temperature. After two wash cycles with PO-buffer [100 mM KH_2_PO_4_, 1.2 M Sorbitol, pH 7,5], cells were resuspended in the same buffer supplemented with 40 µg/ml Zymolyase 100 T (Seikagaku Corp.) and 10 mM ß-Mercaptoethanol, and incubated for 30 min at 25°C. 15 µl of the sample were transferred onto a poly-D-lysine coated slide and incubated at room temperature for 5 min. The slide was then immersed in −20°C cold methanol for 6 min immediately followed by incubation in −20°C cold acetone for 30 sec. The slides were air-dried before the attached cells were rinsed with 2% BSA in PBS for 1 h and incubated with anti-polyQ antibody (1∶100 dilution in 2% BSA in PBS). Cells were washed ten times with 2% BSA in PBS and then incubated with Cy3-conjugated anti-rabbit IgG (1∶1000 dilution in 2% BSA in PBS) in the dark for 1 h. The nuclei were counterstained with Hoechst 33258 (0,2 ng/ml in water) for 10 sec. Cells were viewed under the fluorescence microscope Axioplan-2 (Zeiss).

## Supporting Information

Figure S1 Western blot analysis of yeast cell extracts containing recombinant proteins. GT17 [pin^−^][psi^−^] cells were co-transfected with either PrD/Q52 or PrD/Q90 and transformants were grown for 3 days at 30°C. Cells were lysed and subjected to SDS-PAGE using a modified buffer system previously described (Schägger H (2006) Tricine-SDS-PAGE. Nat. Protoc. 1: 16–22). The expression levels of recombinant proteins were monitored by immunoblotting using an anti-polyQ and an anti-PrD antibody. The polyclonal rabbit anti-PrD antibody was raised against the PrD domain (1-123 aa) of Sup35.
(1.07 MB TIF)

Table S1 Primer pairs used in this study.
(0.04 MB DOC)
