# Supplementary figures and images for: Pathogenic Polyglutamine Tracts Are Potent Inducers of Spontaneous Sup35 and Rnq1 Amyloidogenesis
Source: PLoS One. 2010 Mar 10;5(3):e9642. doi: 10.1371/journal.pone.0009642 (PMC2835767; doi:10.1371/journal.pone.0009642)

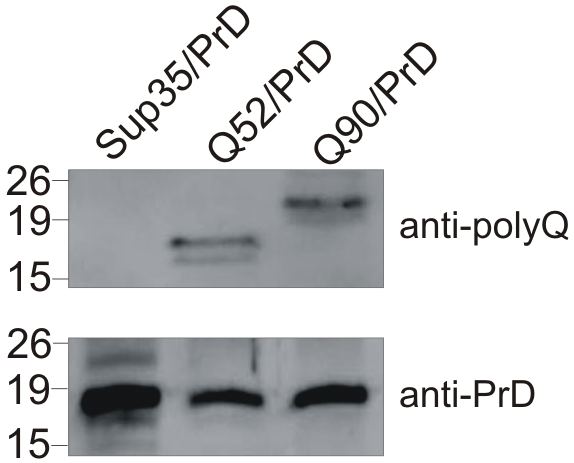

Supplement: Figure S1 — Western blot analysis of yeast cell extracts containing recombinant proteins. GT17 [pin−][psi−] cells were co-transfected with either PrD/Q52 or PrD/Q90 and transformants were grown for 3 days at 30°C. Cells were lysed and subjected to SDS-PAGE using a modified buffer system previously described (Schägger H (2006) Tricine-SDS-PAGE. Nat. Protoc. 1: 16–22). The expression levels of recombinant proteins were monitored by immunoblotting using an anti-polyQ and an anti-PrD antibody. The polyclonal rabbit anti-PrD antibody was raised against the PrD domain (1-123 aa) of Sup35. (1.07 MB TIF) [file pone.0009642.s001.tif]
