# Supplementary material for: Pathogenic Polyglutamine Tracts Are Potent Inducers of Spontaneous Sup35 and Rnq1 Amyloidogenesis
Source: PLoS One. 2010 Mar 10;5(3):e9642. doi: 10.1371/journal.pone.0009642 (PMC2835767; doi:10.1371/journal.pone.0009642)
Supplement: Table S1 — Primer pairs used in this study. (0.04 MB DOC) [file pone.0009642.s002.doc]

| Primer | 5´-Sequenz-3´ |
| --- | --- |
| P01 | ACATGCATGCTTGCATGTCTTTTGCTGGC |
| P02 | acatgcatgcgcggtaccatgaatag |
| P03 | CCGCTCGAGCATGTCGGATTCAAACCAAGGCAAC |
| P04 | ataagaatgcggccgcccataccttgagactgtgg |
| P05 | ATAAGAATGCGGCCGCACTCGGCAATTTTAACAATTTTACC |
| P06 | ATAAGAATGCGGCCGCATGGCGACCCTGGAAAAGCTGATG |
| P07 | GGAATTCTACGGCGGTGGCGGCTGTTGCTG |
| P08 | GGAATTCATGTCGGATTCAAACCAAGG |
| P09 | CGGGATCCTCACATACCTTGAGACTGTGG |
| P10 | GGAATTCGCTCTGCAGAGAGGATCG |
| P11 | CGGGATCCTTACTCGGCAATTTTTAACAATTTTACC |
| P12 | aactgcagagcggccgcatggcgaccctggaaaagctgatg |
| P13 | cccaagcttctacggcggtggcggctgttgctg |
| P14 | CCGCTCGAGCATGTTTGGTGGTAAAGATCACG |
| P15 | GGAATTCATGTCGGATTCAAACCAAGG |
| P16 | ACGCGTCGACAATGGATACGGATAAGTTAATCTC |
| P17 | ATAAGAATGCGGCCGCTCAGTAGCGGTTCTGGTTGCCGT |
| P18 | ATAAGAATGCGGCCGCTGTAGCGGTTCTGGTTGCCGT |
